# Age-Related Patterns of Traumatic Facial Fractures in the Appalachian Tri-state Area: A Five-Year Retrospective Study

**DOI:** 10.7759/cureus.62090

**Published:** 2024-06-10

**Authors:** Armein Rahimpour, Jacy Baxter, Gerard Giangrosso, Abigail Murphy, Paul Bown, David A Denning, Peter Ray, Barry Rahman

**Affiliations:** 1 General Surgery, Marshall University Joan C. Edwards School of Medicine, Huntington, USA; 2 Plastic and Reconstructive Surgery, Marshall University Joan C. Edwards School of Medicine, Huntington, USA

**Keywords:** pediatric facial plastic surgery, facial fractures, orbital fracture, mandibular fracture, craniofacial, appalachian region

## Abstract

Introduction

Traumatic facial injuries, leading to facial fractures represent a significant subset of traumatic events, with age emerging as a crucial determinant influencing both their etiology and outcomes. Understanding the age-related patterns of traumatic facial fractures is essential for developing targeted prevention and management strategies. In this context, the Appalachian tri-state area stands as an underexplored region concerning this issue, necessitating comprehensive research to elucidate the nuances of age-related traumatic facial fractures within this geographic context.

Methods

This retrospective study delves into the age-related patterns of traumatic facial fractures within the Appalachian tri-state area, drawing upon patient records from Cabell Huntington Hospital and Saint Mary's Medical Center spanning a five-year period. The study cohort encompasses 623 patients categorized into three age groups: individuals aged <22 years, those aged 22-65 years, and individuals over 65 years. Data analysis involves meticulous examination of mechanisms of injury, injury severity scores (ISSs), hospital length of stay, and the prevalence of surgical interventions across different age cohorts.

Results

Out of 623 patients, 104 (16.7%) were under 22 years old, 367 (58.9%) were between 22 and 65 years old, and 152 (24.4%) were over 65 years old. The majority were male (70%). Falls were the most common cause of facial fractures in patients over 65 (78%), while assaults were predominant in the 22-65 age group (24%), and motor vehicle collisions (MCVs) in those under 22 (34%). The median ISS and hospital stay durations were similar across age groups. 28% of patients underwent surgery, with significant variation among age groups (p<0.001): 38% for <22 years, 33% for 22-65 years, and 11% for >65 years. Mandibular fractures were more prevalent in younger patients, with rates of 12% for <22 years compared to 5.3% for >65 years. Logistic regression analysis revealed that patients aged 22-65 had 4.10 times higher odds (95% CI=2.38, 7.45, p<0.001) of undergoing surgery, while those under 22 had 5.14 times higher odds (95% CI=2.73, 10.0, p<0.001) compared to those over 65. Significant associations were found for mandibular and bilateral mandibular outcomes in patients aged 22-65 years.

Discussion

These findings underscore the imperative for tailored prevention strategies and age-specific treatment protocols to optimize patient outcomes. Fall prevention initiatives for the elderly and interventions addressing sports-related injuries for younger individuals are paramount. Moreover, the study highlights the necessity of specialized care protocols for elderly patients to minimize hospital stay durations and manage age-related comorbidities effectively. Moving forward, further research should address limitations, validate findings, and explore the efficacy of specific interventions, thereby paving the way for enhanced preventive measures and management strategies tailored to the diverse age cohorts affected by traumatic facial fractures in the Appalachian region.

## Introduction

Facial injuries constitute a significant portion of traumatic events, with facial fractures representing a notable subset of consequences [1-2]. The incidence of facial bone fractures is approximately 3.53 million cases, with a prevalence of about 0.767 million cases worldwide [2]. These fractures have enduring implications, impacting patients across their lifespans [2]. Age emerges as a pivotal determinant in both the etiology and outcomes of facial fractures, delineating distinct patterns across different age groups [1]. Facial fractures exhibit distinct gender disparities, with males experiencing a higher incidence [3]. The incidence of these fractures is infrequent before the age of five but escalates steadily from early schooling to adolescence, with different treatment strategies [3].

Among elderly patients, the increased risk of falls is multifactorial, attributed to factors such as visual impairment, muscle weakness, cardiovascular disease, endocrine dysregulation, and neurological conditions like dementia [4-6]. The prevalence of these conditions rises with advancing age, predisposing older individuals to traumatic facial injuries [3-6]. Conversely, younger demographics are more susceptible to sports-related injuries, assaults, and motor vehicle collisions (MCVs), reflecting the diverse mechanisms underlying facial trauma across age cohorts [7].

Importantly, age significantly influences the outcomes of facial fractures. Older patients often necessitate prolonged hospital stays compared to their younger counterparts, reflecting the complexity of managing injuries in this population and the potential for age-related comorbidities to complicate recovery [5-7]. Studies have shown that the aging population, due to frailty and higher comorbidity rates, often requires more intensive and prolonged medical interventions following trauma, with worse outcomes [8]. This underscores the necessity for specialized treatment protocols tailored to the elderly to enhance recovery and reduce hospital stays.

Despite the evident significance of age in shaping the landscape of traumatic facial fractures, formal studies exploring age-related patterns in specific geographical regions, such as the Appalachian tri-state area, remain scarce. Given the escalating incidence of both pediatric and adult trauma in this region, there is a pressing need for comprehensive research to elucidate the nuances of age-related traumatic facial fractures.

This retrospective study aims to address this critical gap in knowledge by systematically examining the mechanisms and injury patterns of facial fractures across various age groups in the Appalachian tri-state area. By elucidating age-related trends in facial trauma, the findings of this study have the potential to inform healthcare providers, enabling them to better comprehend risk factors and develop more targeted and effective prevention and management strategies tailored to the needs of diverse age cohorts.

## Materials and methods

The study received approval from the Marshall University Institutional Review Board (IRB No. 1991431-1). Patient records were retrospectively reviewed from our trauma registry at Cabell Huntington Hospital and Saint Mary's Medical Center. Both hospitals are academic teaching hospitals, regional referral centers, and American College of Surgeons verified Level-2 Trauma Centers in Huntington, WV.

The analyzed medical files belonged to patients who presented between January 1, 2017, and December 31, 2021 (a period of five years). Data were obtained by contacting the Information Technology (IT) departments at each center. The request included any patient with traumatic facial fractures who presented to the emergency department at the level II trauma centers during the five-year period. We did not exclude any patients. All patients who had at least one facial fracture secondary to trauma were included in this study. Additionally, we requested age, gender, Injury Severity Score (ISS), and hospital length of stay from the IT team. The initial sample consisted of 623 patients. All collected data were centralized using Microsoft Excel software. Medical records for those MVC patients were reviewed, and data on the type of facial fracture (nasal, bilateral nasal, orbital, bilateral orbital, mandibular, bilateral mandibular, maxillary, bilateral maxillary, zygomatic, and bilateral zygomatic) and facial fractures requiring operation were extracted.

In our study, we relied on the Adult Traumatic Life Support (ATLS) framework, a robust training program tailored to equip healthcare providers with the skills needed to effectively manage acute traumas. Our age cutoffs were determined based on compelling evidence indicating that individuals aged 65 and above commonly present with preexisting conditions that significantly impact both morbidity and mortality rates [9]. This demographic has shown to be at a two-fold higher risk of mortality from various traumatic injuries, including blunt and penetrating traumas, hypothermia, and infectious diseases such as tetanus [9]. Hence, individuals aged 65 and older were categorized as belonging to our geriatric/older population. To delineate our pediatric population, we used different pediatric textbooks available [10-12]. Patients under 65 were further divided into two distinct groups: those aged 22 to 65, and those under 22. The primary predictor variable was age group categorized as >65 years, 22-65 years, and <22 years. 

Descriptive statistics were used to summarize sample characteristics. Continuous variables were reported as means ± standard deviations (SD), median, first quartile, and third quartile. Categorical variables were reported as numbers (N) and percentages (%). One-way ANOVA assessed age differences among groups. The Kruskal-Wallis test was used for non-normally distributed continuous variables such as ISS and hospital length of stay. The chi-square test determined significant differences between age groups for each categorical variable, while Fisher’s exact test was used when the expected count was less than five.

Logistic regression analysis assessed the association between age group and operative status and outcomes, adjusting for gender. Odds ratios (ORs) and 95% confidence intervals (CIs) were calculated. All statistical analyses were performed using SAS (SAS 9.4, SAS Institute Inc., Cary, NC, USA). Statistical significance was defined by a two-sided test with a p-value<0.05.

## Results

The study included a total of 623 patients, of which 104 (16.7%) were aged <22 years, 367 (58.9%) were aged 22-65 years, and 152 (24.4%) were aged >65 years (Table 1). The majority of the patients were male (70%), and the gender distribution was significantly different among the age groups (p<0.001). The percentage of males is highest among the patients aged 22-65 years (N=293, 80%), followed by the youngest group (N=68, 65%), and the oldest group (N=73, 48%).

**Table 1:** Sample Characteristics by Age Group. Data presented as n (%), excluding SD. n, number; %, percentage; SD, standard deviation; IQR, interquartile range; MOI, mechanism of injury; MCC, motorcycle crash; MVC, motor vehicle crash; ISS, injury severity score; THD, total hospital days

Variables	Overall (n=623)	Age <22 (n=104)	Age 22-65 (n=367)	Age >65 (n=152)	p-value
Age					<0.001
Mean±SD	46±23	14±5	43±13	78±7	
Median (IQR)	45 (27, 65)	15 (10, 19)	42 (32, 53)	78 (71, 83)	
Gender					<0.001
Female	189 (30)	36 (35)	74 (20)	79 (52)	
Male	434 (70)	68 (65)	293 (80)	73 (48)	
MOI					
Assault	103 (17)	11 (11)	87 (24)	5 (3.3)	<0.001
ATV	44 (7.1)	18 (17)	20 (5.4)	6 (3.9)	
Bicycle	14 (2.2)	8 (7.7)	6 (1.6)	0 (0)	
Blunt Other	47 (7.5)	7 (6.7)	40 (11)	0 (0)	
Fall	195 (31)	14 (13)	63 (17)	118 (78)	
Gun	18 (2.9)	3 (2.9)	12 (3.3)	3 (2.0)	
Knife	1 (0.2)	0 (0)	1 (0.3)	0 (0)	
MCC	55 (8.8)	7 (6.7)	42 (11)	6 (3.9)	
MVC	140 (22)	35 (34)	91 (25)	14 (9.2)	
Unknown	6 (1.0)	1 (1.0)	5 (1.4)	0 (0)	
ISS					0.71
Mean±SD	10±9	11±9	11±9	10±7	
Median (IQR)	8 (5, 14)	8 (5, 14)	9 (4, 14)	6 (5, 14)	
THD					0.74
Mean±SD	6±10	7±10	6±11	6±9	
Median (IQR)	3 (2, 7)	4 (1, 8)	3 (2, 7)	3 (2, 5)	
Operation	177 (28)	40 (38)	120 (33)	17 (11)	<0.001
Nasal	142 (23)	22 (21)	81 (22)	39 (26)	0.61
Bilateral Nasal	181 (29)	26 (25)	110 (30)	45 (30)	0.61
Orbit	240 (39)	39 (38)	148 (40)	53 (35)	0.49
Bilateral Orbit	23 (3.7)	6 (5.8)	14 (3.8)	3 (2.0)	0.31
Mandibular	67 (11)	13 (12)	46 (13)	8 (5.3)	0.042
Bilateral Mandibular	32 (5.1)	8 (7.7)	23 (6.3)	1 (0.7)	0.013
Maxillary	198 (32)	25 (24)	130 (35)	43 (28)	0.051
Bilateral Maxillary	35 (5.6)	7 (6.7)	19 (5.2)	9 (5.9)	0.82
Zygomatic	118 (19)	15 (14)	80 (22)	23 (15)	0.092
Bilateral Zygomatic	3 (0.5)	0 (0)	3 (0.8)	0 (0)	0.75
All Traumas	620 (100)	103 (99)	367 (100)	150 (99)	0.069

The most common mechanism of injury in the study population was falls (N=195, 31%), followed by motor vehicle collisions (N=140, 22%) and assaults (N=103, 17%). The distribution of mechanisms of injury was significantly different among the age groups (p<0.001). Falls were the most common mechanism of injury in the oldest group (N=118, 78%), while assaults were the most common mechanism of injury in patients aged 22-65 years (N=87, 24%). Motor vehicle collisions were the most common mechanism of injury in the youngest group (N=35, 24%) (Table 1).

The median ISS was not significantly different among the age groups (p=0.71). The median ISS was eight (IQR=5-14) for the youngest group, nine (IQR=4-14) for the middle group, and six (IQR=5-14) for the oldest group. The median length of hospital stay was four days (IQR=1-8) for the youngest group, three days (IQR=2-7) for the middle group, and three days (IQR=2-5) for the oldest group. There was no significant difference in the total hospital days among the age groups (p=0.74) (Table 1).

A total of 177 patients (28%) underwent an operation for their facial fractures. The proportion of patients undergoing operation was significantly different among the age groups (p<0.001). The youngest group had the highest proportion of patients undergoing operation (N=40, 38%), followed by the middle group (N=120, 33%) and the oldest group (N=17, 11%) (Table 1).

The distribution of fracture sites was significantly different among the age groups for mandibular fractures (p=0.042) and bilateral mandibular fractures (p=0.013). The youngest group had the highest proportion of bilateral mandibular fractures (N=8, 7.7%), while the oldest group had the lowest proportion (N=1, 0.7%). No significant differences were found among the age groups for the other fracture sites (Table 1).

The logistic regression analysis showed a significant association between age group and operation after adjusting for gender (p<0.001) (Table 2). Compared to patients aged over 65, those aged between 22 and 65 had 4.10 times higher odds (95% CI=2.38, 7.45, p<0.001) of operation, while patients aged under 22 had 5.14 times higher odds (95% CI=2.73, 10.0, p 0.001) of operation.

**Table 2:** Association Between Age Group and Outcomes After Adjusting for Gender. OR, odds ratio; CI, confidence interval

Age Group	Operation		
	OR	95% CI	p-value
Age >65	1	1	Red
Age 22-65	4.10	2.38, 7.45	<0.001
Age <22	5.14	2.73, 10.0	<0.001

Age Group	Nasal			Bilateral Nasal		
	OR	95% CI	p-value	OR	95% CI	p-value
Age >65	1	1	Ref	1	1	Ref
Age 22-65	0.93	0.59, 1.49	0.77	0.95	0.62, 1.47	0.81
Age <22	0.83	0.45, 1.51	0.55	0.76	0.43, 1.34	0.35

Age Group	Mandibular			Bilateral Mandibular		
	OR	95% CI	p-value	OR	95% CI	p-value
Age >65	1	1	Ref	1	1	Ref
Age 22-65	2.58	1.22, 6.16	0.020	9.00	1.82, 163	0.034
Age <22	2.57	1.04, 6.77	0.045	11.8	2.10, 221	0.021

Age Group	Orbit			Bilateral Orbit		
	OR	95% CI	p-value	OR	95% CI	p-value
Age >65	1	1	Ref	1	1	Ref
Age 22-65	1.26	0.84, 1.91	0.26	1.76	0.54, 7.91	0.39
Age <22	1.12	0.66, 1.89	0.67	2.86	0.73, 13.9	0.15

Age Group	Maxillary			Bilateral Maxillary		
	OR	95% CI	p-value	OR	95% CI	p-value
Age >65	1	1	Ref	1	1	Ref
Age 22-65	1.21	0.79, 1.87	0.39	0.75	0.33, 1.83	0.51
Age <22	0.74	0.41, 1.31	0.31	1.06	0.36, 2.96	0.92

Age Group	Zygomatic		
	OR	95% CI	p-value
Age >65	1	1	Ref
Age 22-65	1.21	0.72, 2.09	0.48
Age <22	0.81	0.39, 1.65	0.57

Regarding the specific outcomes, the logistic regression analyses showed only two significant associations after adjusting for gender. For mandibular and bilateral mandibular outcomes, patients aged between 22 and 65 had 2.58 times higher odds (95% CI=1.22, 6.16, p=0.020) and those aged under 22 had 2.57 times higher odds (95% CI=1.04, 6.77, p=0.045) of mandibular outcome, compared to those aged over 65. Patients aged between 22 and 65 also had 9.00 times higher odds (95% CI=1.82, 163, p=0.034) and those aged under 22 had 11.8 times higher odds (95% CI=2.10, 221, p=0.021) of bilateral mandibular outcome, compared to those aged over 65.

No significant association was found between age group and nasal, bilateral nasal, orbit, bilateral orbit, maxillary, bilateral maxillary, or zygomatic outcomes after adjusting for gender (Table 2).

## Discussion

The findings of this study offer valuable insights into the epidemiology and outcomes of traumatic facial fractures across different age groups in the Appalachian tri-state area. The distinct age-related patterns observed underscore the necessity for tailored prevention and management strategies to address the unique needs of each age cohort.

Age-related mechanisms of injury

The study corroborates existing literature indicating that the mechanisms of facial fractures vary significantly by age. Among elderly patients, falls were the predominant cause of facial trauma, aligning with previous research that links this demographic to a higher susceptibility to falls due to underlying factors such as visual impairments, muscle weakness, and neurological conditions like dementia [5-6]. This highlights the critical need for fall prevention programs and interventions aimed at mitigating risk factors in the elderly population.

In contrast, younger patients were more frequently injured due to sports-related activities, assaults, and MCVs. This finding is consistent with studies that have identified these activities as common causes of facial trauma in younger demographics [4]. Research has further emphasized the need for targeted preventive measures, such as the use of protective gear in sports and safe driving campaigns, to reduce the incidence of facial fractures in younger populations [13,14].

Influence of age on outcomes

Our study also demonstrates that age significantly influences the outcomes of facial fractures. Elderly patients exhibited longer hospital stays, attributed to the increased complexity of managing injuries in this age group due to comorbidities and age-related physiological changes [4-6]. This underscores the importance of comprehensive care plans for healthcare providers. Similar findings have been reported in other studies, indicating that the aging population requires specialized care protocols to manage the higher risk of complications and longer recovery periods [4-6].

The study's findings shed light on a notable trend: younger patients were more prone to undergoing surgical intervention than their older counterparts. This observation may stem from various factors, encompassing the nature of injuries sustained by younger individuals and potential differences in clinical strategies or patient inclinations. Younger individuals are likelier to experience facial fractures due to high-energy mechanisms, such as MVCs. These incidents frequently lead to intricate fractures that necessitate surgical treatment, a contrast to the most common cause of facial fractures in the elderly group, falls. This dichotomy underscores the importance of recognizing diverse injury mechanisms across age groups and tailoring treatment approaches accordingly. Second, facial fractures in younger patients may involve the developmentally active facial skeleton, necessitating precise anatomical reduction to prevent long-term functional and aesthetic sequelae. Additionally, younger patients may have a higher tolerance for surgical procedures and anesthesia compared to older individuals, enabling surgeons to pursue more aggressive treatment approaches to achieve optimal outcomes. Overall, the higher rate of operative treatment in younger patients underscores the importance of tailored management strategies to address the unique needs of different age cohorts in the management of traumatic facial fractures.

Implications for clinical practice and policy

The differential patterns of injury and outcomes across age groups have significant implications for clinical practice and health policy. For the elderly, there is a pressing need for enhanced fall prevention strategies and interventions tailored to reduce the risk of fractures. Prevention efforts for younger patients should focus on mitigating risks associated with high-impact activities such as sports and driving. Public health campaigns promoting the use of protective equipment in sports and safe driving practices could play a pivotal role in reducing the incidence of facial fractures in this group.

Additionally, our findings highlight the importance of age-specific treatment protocols. Given the higher likelihood of surgical intervention in younger patients, clinicians should be prepared to address the unique surgical and postoperative needs of this population. Conversely, the management of elderly patients should prioritize minimizing hospital stay durations through effective rehabilitation and management of comorbidities.

Research limitations and future directions

While this study provides significant insights, it is not without limitations. The retrospective nature of the data collection may introduce biases related to the accuracy and completeness of medical records. Additionally, the study is geographically limited to the Appalachian tri-state area, which may limit the generalizability of the findings to other regions with different demographic and healthcare characteristics.

Future research should aim to conduct prospective studies with larger and more diverse populations to validate and expand on these findings. Additionally, exploring the impact of specific interventions on the prevention and management of facial fractures across different age groups could provide promising insights for improving patient outcomes.

## Conclusions

In summary, our retrospective review examined the relationship between safety device utilization and facial fractures in MVC patients within the Appalachian tri-state area. Contrary to some prior research findings, our study did not observe significant associations between the use of seatbelts, airbags, or both, and the occurrence of facial fractures. While safety devices remain crucial components of injury prevention efforts, the complexity of MVC-related injuries suggests that a multifaceted approach is necessary to address this public health concern effectively.

Moving forward, it is imperative to continue investigating the interplay between safety measures, injury mechanisms, and patient outcomes in MVCs. By leveraging insights from ongoing research and adopting comprehensive strategies that encompass vehicle safety technologies, public awareness campaigns, and trauma care protocols, we can strive to reduce the burden of MVC-related injuries on individuals and communities.
